# Supplementary material for: Understanding the dynamics of obesity prevention policy decision-making using a systems perspective: A case study of Healthy Together Victoria
Source: PLoS One. 2021 Jan 22;16(1):e0245535. doi: 10.1371/journal.pone.0245535 (PMC7822316; doi:10.1371/journal.pone.0245535)
Supplement: S1 Appendix — (DOCX) [file pone.0245535.s001.docx]

**S1 Appendix. Number of interview participants and data collection timeframe for each Healthy Together Victoria policy study.**

| **HTV policy study** | **Number of interviewees** | **Timeframe** |
| --- | --- | --- |
| *Achievement Program* | 10 | December 2015 - October 2016 |
| *Jamie’s Ministry of Food* | 6 | December 2015 - October 2016 |
| *LiveLighter*® | 11 | December 2015 - November 2016 |
| *Menu Kilojoule Labelling Legislation* | 13 | August 2016 - April 2017 |
| *Land Use Planning Policies* | 24 | November 2015 - April 2017 |
| *Healthy Catering Policies* | 11 | December 2015 - April 2017 |
