# Supplementary material for: Understanding the dynamics of obesity prevention policy decision-making using a systems perspective: A case study of Healthy Together Victoria
Source: PLoS One. 2021 Jan 22;16(1):e0245535. doi: 10.1371/journal.pone.0245535 (PMC7822316; doi:10.1371/journal.pone.0245535)
Supplement: S2 Appendix — (PDF) [file pone.0245535.s002.pdf]

S2 Appendix - Semi-structured interview guide

Warm up question:

1. Can you tell me a bit about your role in regard to [Healthy Together Victoria policy]?

Key questions:

2. Why do you think the policy was [or was not] able to be adopted?
3. Now I'd like to talk about some of the broader contextual factors that influenced the decision-making related to the policy. Can you describe the key influences on the policy decision-making processes from your perspective? Prompts: Can you say more about that? Can you give me an example? Can you tell me a bit more about that?
4. Can you please reflect on any relevant broader political factors (e.g., free market versus government intervention) and how these influenced the policy, if at all?
5. How did timing influence in the policy process, if at all?
6. How was evidence used throughout the policy process, if at all?
7. I've observed many institutional factors that can influence policy (such as inter-departmental collaboration, role and involvement of other departments, role of things like treasury/budget reviews etc.) can you please comment on how these played a role in the adoption of this policy, if at all?
8. Can you please reflect on the role of different groups and networks in the policy process?
9. Can you please reflect on the values and beliefs of key or influential individuals?
10. Can you please reflect how the issue of obesity is framed (e.g., personal responsibility or environmental/ governmental responsibility), and how this played a role, if at all?

Other general reflections:

11. What do you think policy actors (i.e., advocacy groups, community organisations, local government) can learn to influence policy in support of obesity prevention?
12. To assist with my data collection can you please:
    a. Suggest any other individuals I should talk with to get insight into this policy development process?
    b. b. Suggest appropriate documents that would be useful for me to include into my dataset?
