# Supplementary material for: Understanding the dynamics of obesity prevention policy decision-making using a systems perspective: A case study of Healthy Together Victoria
Source: PLoS One. 2021 Jan 22;16(1):e0245535. doi: 10.1371/journal.pone.0245535 (PMC7822316; doi:10.1371/journal.pone.0245535)
Supplement: S3 Appendix — (DOCX) [file pone.0245535.s003.docx]

**S3 Appendix. Documents collected and analysed within each Healthy Together Victoria policy study.**

| **Document type** | **Document description** | **Number of documents** | | | | | | | | |
| --- | --- | --- | --- | --- | --- | --- | --- | --- | --- | --- |
|  |  | ***Achievement Program*** | ***Jamie’s Ministry of Food*** | ***LiveLighter***® | ***Menu Kilojoule Labelling Legislation*** | ***Land Use Planning Policies*** | | | ***Healthy Catering Policies*** | |
|  |  |  |  |  |  | **State level** | **Local level** | | **State level** | **Local level** |
| Internal policy briefings and memos, proposals | Short summaries of what is known about a particular issue and potential policy options designed to facilitate decision-making; records of meetings | 7 | 14 | 8 | - | 10 | 20 | | 17 | 12 |
| Consultation and discussion papers | Government publications developed for the purposes of stakeholder or public discussion and comment. These aimed to facilitate discussion regarding policy design, scope and implementation planning | 9 | - | - | 1 | 2 | | | 4 | 3 |
| Victorian Hansard | The official verbatim record of debates of the Victorian Parliament | - | 3 | 1 | 6 | 1 | | | 4 | |
| Media reports | Any media report that referred directly to the HTV policy processes | - | 5 | 40 | 53 | 215 | | | - | |
| Observation notes | Open ended narrative field notes on daily activities related to the Menu Kilojoule Labelling legislation | - | - | - | 3 | - | | | - | |
| Other | Evaluation reports, communication reports including campaign reports, stake evidence reports and other government papers | 1 | 5 | 20 | 2 | - | | | - | |
| Land Use Planning policies | At the state government level the Plan Melbourne document and Local Government Planning schemes were reviewed 2011-2017 | - | - | - | - | 1 | | 4 | - | |
| Public submissions to policy proposals | Publically available submissions to the Plan Melbourne policy proposal. Only those from the latest proposal (2017) were available | - | - | - | - | 107 | | | - | |
| Event logs | HTC practitioner reflective practice journal entries | - | - | - | - | 4 | | | 3 | |

HTV: Healthy Together Victoria, HTC: Healthy Together Communities (implementation sites of HTV)
