# Supplementary material for: Understanding the dynamics of obesity prevention policy decision-making using a systems perspective: A case study of Healthy Together Victoria
Source: PLoS One. 2021 Jan 22;16(1):e0245535. doi: 10.1371/journal.pone.0245535 (PMC7822316; doi:10.1371/journal.pone.0245535)
Supplement: Attachment — Submitted filename: HTV CLD Response to Reviewers 201218 final.docx [file pone.0245535.s004.docx]

| **Reviewer comment** | | **Authors’ response** | **Changes made to text** |
| --- | --- | --- | --- |
| Reviewer 1 | | | |
| 1. | One concern that I have is related to the data analysis. Data was collected by one of the researchers and it seems that this researcher also analysed all data and produced the diagrams. It is not clear how reliability of the analysis was assessed. It would have been good to have a second coder code/analyse the same data to prevent researcher bias. | All data analysis was initially performed by one of the researchers (BC), and reviewed by another (GS), with queries resolved in consultation with a third researcher (BS) where necessary.  We employed a number of strategies to help minimise potential research bias related to the centralised data collection and analysis approach. These included using deductive analysis and a pre-defined codebook, triangulation of data across methods (e.g., interviews, documents and field notes) and data sources (e.g., multiple participants and policy processes), and the use of reflexive practices throughout the study.  We have now amended the methods section to provide more details of who was involved in data analysis. We have also added more details to the limitations section to discuss the risk and control of researcher bias related to the analysis of data. | **Methods (Page 11, Line 220-2):**  All data analysis was initially performed by one of the researchers (BC), and reviewed by another (GS), with queries resolved in consultation with a third researcher (BS) where necessary.  **Strengths and limitations (Page 31-32, Line 719 – 25):** In regards to limitations of the study, data collection and initial analysis was conducted by one researcher (BC), which has the potential to introduce researcher subjectivity and bias. Several strategies were used to reduce potential bias, including review of analysis outputs by a second researcher, using a pre-defined deductive codebook, triangulation of data across methods (e.g., interviews, documents and field observations) and data sources (e.g., multiple participants and policy processes), and utilisation of reflexive practices throughout the study [103]. |
| 2. | Figure 1 displays the way the data was analyzed, but it is unclear whether some findings are based on the individual analyses that are reported elsewhere or new analyses were done on the data. | We have revised the methods section to clarify that the findings are based on themes that were consistent across the individual analyses, and that these themes were identified by using a two stage process whereby the first stage involved analysis and development of a CLD for each HTV initiative, and the second stage involved synthesising the CLDs to identify cross-cutting themes. | **Methods (Page 11, Line 216-24):** A two-stage analysis process was undertaken. In the first stage, data were analysed for each of the selected policy interventions separately. As part of this first stage of analysis, a CLD was generated to describe the dynamics of the policy process for each intervention. In the second stage, the six individual CLDs were synthesised to produce a meta-CLD of the HTV policy system based on themes that were consistent across the six CLDs. All data analysis was initially performed by one of the researchers (BC), and reviewed by another (GS), with queries resolved in consultation with a third researcher (BS) where necessary. Further details of each stage of the analysis are provided below and a schematic overview of the study analysis approach is displayed in **Fig 1**. |
| 3. | Results of the study are extensively discussed. I miss a bit more concrete translation into recommendations for future policy implications. It is now described at a bit abstract level. | We have now added a new section in the discussion on policy implications and recommendations. | **Discussion (Page 29-31, Line 671 – 713):** **Policy implications and contributions to the literature**  This study builds upon previous obesity prevention policy literature by providing ‘real-world’ insight into the leverage points and trade-offs that need to be considered by those seeking to advance obesity prevention policy implementation. Several barriers to obesity prevention policy adoption have previously been identified, including powerful food industry lobbying, limited political skills and knowledge among the public health community, and institutional factors such as government ‘silos’ and political turnover [46, 96]. To date, however, few studies have investigated the dynamics between the various influences on policy progress [37, 97].  A key insight identified in the current study was the importance of obesity policy actor skills and capability, specifically the ability to develop and propose desirable policy solutions. While improved policy actor capability has also been recommended in previous obesity prevention policy studies [37, 96, 98], there remains a lack of empirical evidence regarding the skills on which to focus capacity building efforts. The current study goes some way to addressing this gap by having elucidated specific skills likely to be important, including the ability to frame policy solutions to decision-maker beliefs and broader government objectives, the skills to effectively engage stakeholders, and the utilisation of evidence to develop viable policy solutions. Nevertheless, further research is required to explore effective ways to enhance these skills within the obesity prevention context.  The study also highlighted the potential trade-offs [61] regarding various policy processes (e.g., whole-of-government processes and stakeholder engagement) that are typically cited as ‘best practice’ [72, 99]. The study demonstrated that while extensive stakeholder engagement helped to increase support for policy proposals, stakeholder engagement also introduced a risk to the timeliness and effectiveness of policy adoption by amplifying a number of divergent stakeholder views. Policy makers should be cognisant of this tension when designing stakeholder engagement processes, and consider appropriate structures and processes to effectively and efficiently engage stakeholders. For example, targeted or hybrid consultation approaches [100] could be considered, along with the establishment of clear parameters and processes for responding to feedback [70, 77].  In addition, this study provided a nuanced view of the potential role of evidence as part of policy processes. Although policy makers, advocates and previous policy studies agree on the importance of evidence in obesity prevention policy, the focus of such discussion has largely been on evidence of the policy problem and effectiveness of policy solutions [18, 97, 101]. This study demonstrated the need for policy actors to employ evidence regarding multiple aspects of policy implementation in support of policy proposals. For example, evidence of feasible implementation of policy from other jurisdictions and related contexts was found to be valuable in increasing decision-maker acceptability and support. |
| Reviewer 2 | | | |
| 1. | The topic of these research is obesity prevention policy adoption. The paper is very well and clearly written with the problem and issues clearly stated. The topic of obesity, its elements, causes and current management challenges are not addressed. The topic of obesity appears to be incidental to the subject at hand, which is an abstract exploration of the application of 3 political theoretical frameworks to documentation and qualitative stakeholder interview data relating to obesity prevention policy. Measured core elements of the analysis are not elucidated so are difficult to evaluate. The method itself is difficult to evaluate and is not replicable in its current form. The results do not report the qualitative stakeholder interview data, and instead provide a summary of findings, which is difficult to assess. The conclusions are not supported by sufficient data reporting. | Thank you for your review of this manuscript. We have addressed each of your detailed comments below. | See changes to specific comments below. |
| 2. | Introduction: The paper identifies and characterises system feedback processes using a case study design. Focus is largely on policy methods without explaining the complexity of the obesity issue per se. The paper would benefit from provision of more detail in this respect | We have expanded the introduction to discuss the issues and complexity of obesity and relation to obesity prevention policy. | **Introduction (Page 4, Line 50 – 55):** The causes of obesity are complex, with numerous individual, social and environmental factors identified as playing a role in the etiology of overweight and obesity [3, 4]. While there is some debate among stakeholder groups as to the most appropriate response to rising rates of obesity [5, 6], it is generally agreed that comprehensive, multi-sectoral approaches, across multiple levels of government, are required [7, 8]. |
| 3. | Methods: Interview sample size is robust, however the sample itself is not described in any detail. | We have updated the methods section to provide further details about the sample of interview participants. | **Methods (Page 9-10, Line 171 – 83):** Participants included: current and former politicians (n=4); senior ministerial staff (n=2); government officials at state (n=26) and local levels (n=12); academics (n=4); and senior representatives from key public health organisations (n=7) and private sector organisations (n=2), such as food retailers. All but one of the potential interviewees who were invited to participate accepted in relation to each of *The Achievement Program, Jamie’s Ministry of Food*, *LiveLighter®* and *Healthy Catering Policies* investigations, whilst two identified interviewees did not participate in relation to *Menu Kilojoule Labelling Legislation* and *Land Use Planning Policy* (n=6 in total). There were also a small number of potential interviewees (n=4), identified through the snowball sampling process, who were not contactable in relation to *The Achievement Program*, *Jamie’s Ministry of Food* and *Menu Kilojoule Labelling Legislation*, as they had either left their respective organisations or were on extended leave at the time of data collection. |
| 4. | Methods: Snowball sampling is used, however this is not a rigorous approach to sampling, and  while the various types of stakeholders are listed, no information is provided on the target samples for each type of stakeholder, if or how a balance of perspectives was sought, and how participants were selected beyond the fact that (1) snowball sampling was undertaken, and (2) selection was based on ability to provide detailed insight etc. How was ability assessed? How were stakeholder type numbers decided upon? Within what bounds? How long did the interviews last? Where did they take place? (How) were they recorded? How many researchers conducted the interviews? What steps were taken to standardise across researchers? How were the questions developed? | We have revised the methods section to make it clear that the primary sampling method was purposive sampling, which was supplemented by snowball sampling. We have also clarified that adequacy and appropriateness of participants was facilitated by the positioning of one researcher in the policy context as part of a research placement within the Victorian Department of Health and Human Services. This research design allowed the identification of relevant stakeholders to be assessed based on observations and discussion with policymakers within the policy context.  We note that triangulation of interview data with relevant documents and observations also helped to enhance reliability of the findings.  In relation to your comments regarding details related to various aspects of the interviews, we have revised the methods section to provide additional details and address any gaps. | **Methods (Page 9, Line 160 - 71):** Key informant interviews were conducted between December 2015 and April 2017. Participants were identified through purposive sampling, supplemented by snowball sampling [45]. Purposive sampling was facilitated by the positioning of one of the researchers (BC) in the policy context, which helped with identification of relevant and appropriate interview participants through the researcher’s direct observations and interactions with policy makers. Participants were selected based on their anticipated ability to provide detailed insight and first-hand experience into the policy processes related to each of the included HTV policy interventions (assessed separately for each policy). The total number of interviewees conducted in relation to each of the HTV policy interventions ranged from six (*Jamie’s Ministry of Food*) to 24 (*Land Use Planning Policies*)**.**  The varying number of interviewees for each policy reflects the differing scale, prominence and number of stakeholders involved in the policy processes regarding each of the HTV initiatives.  **(Page 9-10, Line 184 - 9):** Interviews were guided by a semi-structured interview schedule (**S1 Appendix**), which was informed by a review of the literature and the political science frameworks underpinning the study [46]. Participants selected the location of the interviews, which occurred either within a workplace or neutral public setting (e.g., café), except for four participants who elected to participate by phone interview. The duration of interviews ranged from 16 minutes to 75 minutes. |
| 5. | Methods: There appears to be an inconsistent balance in the number of interviews around the 6 HTV initiatives, why was this? How will this affect the balance of perspectives? A wide and inconsistent range of documents are included, how will this affect the consistency of the findings? | The varying number of interviewees and documents analysed reflects the differing scale, prominence and number of stakeholders involved in the policy processes regarding each of the HTV initiatives. As per our previous comment, appropriateness and adequacy of interview participants was facilitated by the researcher’s positionality within the policy context.  Nevertheless, we recognise that the study may have been limited by the inaccessibility of some potential interviewees, such as politicians and political advisors, and some relevant documents. We have addressed this limitation within the discussion section. | **Methods (Page 9, Line 160 - 71):** Key informant interviews were conducted between December 2015 and April 2017. Participants were identified through purposive sampling, supplemented by snowball sampling [45]. Purposive sampling was facilitated by the positioning of one of the researchers (BC) in the policy context, which helped with identification of relevant and appropriate interview participants through the researcher’s direct observations and interactions with policy makers. Participants were selected based on their anticipated ability to provide detailed insight and first-hand experience into the policy processes related to each of the included HTV policy interventions (assessed separately for each policy). The total number of interviewees conducted in relation to each of the HTV policy interventions ranged from six (*Jamie’s Ministry of Food*) to 24 (*Land Use Planning Policies*). The varying number of interviewees for each policy reflects the differing scale, prominence and number of stakeholders involved in the policy processes regarding each of the HTV initiatives.  **Methods (page 10-11, Line 203-5)**:  As with the interviews, the varying number of documents analysed for each policy reflects the differing characteristics and scale of the policy processes regarding each of the HTV initiatives.  **Discussion (Page 31, Line 725-33):**  The substantial number of interviews that were conducted, and the high participation rate from potential interviews further increased the reliability of the study. Nevertheless, the inaccessibility of some potential interviewees, including politicians and political advisors, may have limited the perspectives gained. Future studies that successfully engage a greater number of political actors would be particularly valuable. While the study also included a large number of documents as part of the analyses, with access to relevant documents facilitated by the placement of one of the researchers within DHHS, some relevant documents were not available to the researchers as they were classified as ‘in-confidence’ or were held by other government departments. |
| 6. | Three political science theories are selected, however it is not clear how they are used in classifying data to develop key constructs. The reader is referred to another paper (Kim and Andersen) however it would be helpful if a brief explanation was to be given here. | We have provided additional details in the methods related to the process of coding data against the key constructs of the selected political science theories. | **Methods (Page 12, Line 237 – 45):** Data (interviews, documents and field notes) were systematically examined and coded against key constructs of the selected theories using a defined codebook (available on request). Data coding was facilitated by the qualitative software NVivo10. Then, as described by Kim and Andersen [34], coded data were ‘micro-analysed’ to identify cause and effect variables, and the relationships between variables. This process involved iteratively documenting variable names, direction of linkages between elements and the association direction (i.e., polarity). These causal structures were used to develop the final graphical representation of the CLDs using Vensim software (Ventana Systems, Harvard, MA). The findings of the individual analyses are reported elsewhere [49-52]. |
| 7. | Results: These are reported logically and described clearly, however the findings appeared very abstract with no specific supporting evidence provided (e.g. information source in each case: interview or documentation). Information is reported for what was found, however it is not clear how it was found. While the narrative is interesting, it is not clear how the findings were arrived at. | We note that the aim of the study was to holistically identify the underlying drivers and dynamics of obesity prevention policy commitment using system dynamics. The value of system dynamics is the ability to conceptualise complex systems, such as policy processes, as simplified heuristics and provide insight into the opportunities to intervene in such systems. For this reason, the findings report themes that were consistent across multiple HTV initiatives and may appear abstract since there is some generalisation across the analysed policies.  However, in response to your feedback, we have revised the methods section to make the description of the analysis and process for establishing key findings clearer.  We have also now revised each part of the Results section to more clearly indicate the source of the findings. We have also now included verbatim quotes and document excerpts to illustrate and support the meta-CLD findings, throughout the results section.  Finally, we have added a section on policy implications to the discussion section to ensure the paper provides tangible lessons for readers. | **Methods (Page 11, Line 216 – 20):** A two-stage analysis process was undertaken. In the first stage, data were analysed for each of the selected policy interventions separately. As part of this first stage of analysis, a CLD was generated to describe the dynamics of the policy process for each intervention. In the second stage, the six individual CLDs were synthesised to produce a meta-CLD of the HTV policy system based on themes that were consistent across the six CLDs.  **Methods (Page 12, Line 237 – 245):** Data (interviews, documents and field notes) were systematically examined and coded against key constructs of the selected theories using a defined codebook (available on request). Data coding was facilitated by the qualitative software NVivo10. Then, as described by Kim and Andersen [31], coded data were ‘micro-analysed’ to identify cause and effect variables, and the relationships between variables. This process involved iteratively documenting variable names, direction of linkages between elements and the association direction (i.e., polarity). These causal structures were used to develop the final graphical representation of the CLDs using Vensim software (Ventana Systems, Harvard, MA). The findings of the individual analyses are reported elsewhere [47-50].  **Results (various locations throughout):** Reference has now been made in various locations to the specific evidence source (e.g., interviews, documentation) when reporting findings.  **Results (Page 14-15, Line 310 – 22):** This strategic alignment of policies to decision-maker beliefs acted to increase support for policy intervention from decision-makers, in turn, leading to increased adoption of obesity prevention policies. Critically, the level of capability and use of these strategic skills by policy actors increased with each successful policy adoption over time, thus creating a virtuous reinforcing loop. For example, in interviews, policymakers reflected on how during the development of *Menu Kilojoule Labelling Legislation* they learnt to adapt policy framing to align with decision-maker beliefs and increase decision-maker acceptability.  “…*The framing around the policy did get a bit more focused in this third [iteration of the policy] around providing Victorians with informed choice. So that kind of wording was quite strong in this government’s, in this Minister’s speaking. So that was a kind of transformation of the framing*  (DHHS Senior Policy Advisor 1)  **Results (Page 15, Line 324 – 33):** This alignment of policy proposals to other government objectives was an important driver of decision-maker support for several HTV policy interventions. For example, *Jamie’s Ministry of Food* was framed in policy documentation as aligning with broader preventive health policies, such as the Victorian Public Health and Wellbeing Plan, and government objectives related to increased productivity and reduced healthcare costs.  *“There is no ‘silver bullet’ to reducing the prevalence of obesity. JMoF [Jamie's Ministry of Food] can work alongside other government and non-government health initiatives and add value.”*  (DHHS policy documentation, 2011)  **Results (Page 16, Line 349 – 59):** Consequently, according to interview participants, stakeholder consultations reduced the perceived political risks associated with policy adoption, leading to increased support from decision-makers, and thus creating a reinforcing loop (R3). Indeed, a former Minister identified stakeholder consultation as a critical facilitator in the adoption of the *LiveLighter*® campaign.  *“I think part of [the reason that the policy was adopted] was the process of consultation itself, actually. Often people then feel involved, and they have a deeper understanding of what’s going on, what’s trying to be achieved, any potential negatives and how you might manage those. I think [the facilitating factor] was doing [the consultations]”*  (Former Minister 1)  **Results (Page 17, Line 378 – 87):** According to participants, evidence from evaluations of implementation elsewhere in Australia was used to demonstrate the likely positive impact and increase acceptability of several HTV policies. For example, evidence of the impact of the *LiveLighter*® campaign in Western Australia was reported to have helped convince decision-makers that the policy was likely to be effective in Victoria..  *“We’ve seen the [Western Australia] material [for LiveLighter®], and we’re able to adapt that… There was good research behind [the LiveLighter® policy] to suggest that it may well be effective”*  (Former Minister 1)  **(Results, Page 17-18, Line 388 – 401):** In addition, *evidence of implementation feasibility* (R6), often independent of evidence of policy effectiveness, was noted as critical in regards to several policy interventions, including the *Achievement Program,* *Jamie’s Ministry of Food*, *Live Lighter*®, and *Menu Kilojoule Labelling Legislation* [47, 48, 50]. An example of the use of evidence of implementation feasibility was in relation to *Menu Kilojoule Labelling Legislation*, where government policymakers introducing the policy emphasised the established nature of similar policies in other Australian jurisdictions.  *“Kilojoule labelling laws have been in operating for nearly five years now in New South Wales. The scheme is well understood and accepted by industry. Similarly, we expect a smooth transition to the laws in Victoria. It is estimated that 80 per cent of the businesses required by the legislation to display kilojoule content area already doing so in other states. Therefore, the measurements have already been calculated and will be easily available”*  (Victorian Parliament Hansard, Legislative Council, 13 October 2016)  **Results (Page 19, Line 416 – 26):** There was strong evidence from interviews for this effect across the HTV policy process investigations, with examples of internal government staff members facilitating network coordination and negotiation processes with stakeholders to reduce resistance to policy options. In the adoption of the *Achievement Program*, policymakers commented on how they strategically engaged stakeholders from the Department of Education early and throughout the policy process in order to increase their support.  *“[The] original steering group only had two representatives from the Department of Health and eight from Department of Education. We deliberately sought to shift some of that power to Education as a strategy to engage them, to give them a bit more power and control over the direction to inform it right from the beginning”*  (DHHS Senior Policy Advisor 2)  **Results (Page 20, Line 440 – 52):** This risk aversion was particularly evident for the more controversial *LiveLighter*® social marketing campaign and the *Menu Kilojoule Labelling Legislation* whereby additional consultation or modified policy proposals were required to overcome perceived risks [47, 50]. In relation to the *Land Use Planning Policies*, interview participants reflected on how organisational risk aversion increased reluctance to attempt policy changes unless they were aligned with decision-maker attitudes.  *“We were going to do a policy around limiting fast food outlets and put in a Land Use Planning Policy review but we felt that [the local government area] wasn’t really ready to listen to that… because what’s happened in the past was that things have tried to get into the review of Land Use Planning Policy, even around food, and it’s been ‘chucked out’. It has got up to the council and a particular Councillor said “you can’t restrict that sort of stuff” and took it out”*  (Local Government Manager 1)  **Results (Page 21, Line 467 – 79):** Interviewees indicated that stakeholder consultation processes resulted in substantially increased time required for policy change, which, as identified earlier (B1), presented a risk to policy adoption. For example, in the case of the *Land Use Planning Policies*, it was reported that the requirement for public exhibition and consultation increased the complexity of policy change and likelihood of delayed policy decisions.  *“The [strategic planning team] are still dealing with the [public] submissions they’ve had from the [land use planning policy] exhibition, and I think they’re trying to avoid going to Panel [a planning panel hearing - a formal process whereby a planning authority reviews proposed amendments]. The trouble with planning scheme amendments is that if one person makes a submission and they won’t withdraw it – even if it’s irrelevant, you can end up at a panel hearing”*  (Local Government Policy Officer 1) |
| 8. | Given the central importance of capacity and capability of policy makers to the arguments summarised in the discussion, how was capacity and capability of policy makers characterised by participants? Results refer to ‘Government policy makers or external policy actors with sufficient capability were able to frame policy narratives to align policy proposals to decision-maker beliefs’ – how was this evaluated? It is not clear how these inferences were arrived at given the scope and content shown in the Appendices. | A central concept in the MST is that of policy entrepreneurs, which are individuals, coalitions, or organisations that employ various resources and strategies to progress particular policy solutions. The ACF recognises a similar strategic policy actor, policy brokers, who seek to influence policy change. Our study defined (based on the MST and ACF literature) and applied these concepts in our analysis to understand policy actor capability and capacity.  We now recognise that ‘sufficient’ capability in the study context is subjective and it is more appropriate to discuss the policy actor use and capability to employ various strategic tactics. We have revised the text related to policy actor capability and capacity in the results and discussion section accordingly. | **Results (Page 14, Line 301 – 6):** Government policy makers and external policy actors that were able to frame policy narratives to *align policy proposals to decision-maker beliefs* (R1) played an important role in reinforcing policy change. According to interview participants, these actors commonly framed policies as part of a broader suite of complementary initiatives under HTV, particularly where there was criticism of the likely impact of a single policy.  **Results (Page 15, Line 313 – 4):** Critically, the level of capability and use of these strategic skills by policy actors increased with each successful policy adoption over time, thus creating a virtuous reinforcing loop.  **Results (Page 15, Line 323 – 4):** A related reinforcing loop was the *alignment of policy proposals to* *other government objectives* (R2) by policy actors.  **Discussion (Page 24, Line 530 – 3):** Policy actor capability to develop convincing policy proposals increased over time as policy actors gained experience in advocating for change and learned from other jurisdictions that had implemented obesity prevention policies.  **Discussion (Page 24, Line 545 – 50):** The findings of this study highlighted the many policy system feedback loops that are influenced by the skills and capability of policy makers, both of which are central elements within the ACF and MST. When policy actors employed various strategic skills, a number of virtuous system effects were stimulated, such as the development of viable policy solutions that aligned with decision-maker beliefs and government objectives, as well as improved implementation feasibility and stakeholder support. |
| 9. | **Discussion and Conclusion:** Extensive and interesting, however I do not understand how the conclusions were reached given the sparse nature of information provided concerning the methodology. While the case study used related to obesity prevention, there is little in this section which relates to the challenges of obesity prevention, or how obesity prevention differs from other public health issues. This section therefore comes across as very generic, e.g. referring to public health policy in general as opposed to obesity prevention in particular. Previous studies are mentioned, but little information is provided as to how the current findings relate to these earlier studies. Conclusions are difficult to assess given the lack of detail provided relating to the underlying data, and there is insufficient evidence to suggest that the research has been conducted rigorously. | As noted in response to your earlier comments, we have added further details in relation to several aspects of the methods. We trust that these changes now provide sufficient information to assess the study discussion and conclusions.  We have also revised various aspects of the discussion,, including a new section on policy implications to provide greater insights and lessons related to obesity prevention policy, and relevance of previous obesity policy process studies. | **Discussion (various locations):** inserted the term ‘obesity prevention’ in various locations to make clear the relevance to obesity.  **Discussion (Page 29-31, Line 671 – 713):** **Policy implications and contributions to the literature**  This study builds upon previous obesity prevention policy literature by providing ‘real-world’ insight into the leverage points and trade-offs that need to be considered by those seeking to advance obesity prevention policy implementation. Several barriers to obesity prevention policy adoption have previously been identified, including powerful food industry lobbying, limited political skills and knowledge among the public health community, and institutional factors such as government ‘silos’ and political turnover [46, 96]. To date, however, few studies have investigated the dynamics between the various influences on policy progress [37, 97].  A key insight identified in the current study was the importance of obesity policy actor skills and capability, specifically the ability to develop and propose desirable policy solutions. While improved policy actor capability has also been recommended in previous obesity prevention policy studies [37, 96, 98], there remains a lack of empirical evidence regarding the skills on which to focus capacity building efforts. The current study goes some way to addressing this gap by having elucidated specific skills likely to be important, including the ability to frame policy solutions to decision-maker beliefs and broader government objectives, the skills to effectively engage stakeholders, and the utilisation of evidence to develop viable policy solutions. Nevertheless, further research is required to explore effective ways to enhance these skills within the obesity prevention context.  The study also highlighted the potential trade-offs [61] regarding various policy processes (e.g., whole-of-government processes and stakeholder engagement) that are typically cited as ‘best practice’ [72, 99]. The study demonstrated that while extensive stakeholder engagement helped to increase support for policy proposals, stakeholder engagement also introduced a risk to the timeliness and effectiveness of policy adoption by amplifying a number of divergent stakeholder views. Policy makers should be cognisant of this tension when designing stakeholder engagement processes, and consider appropriate structures and processes to effectively and efficiently engage stakeholders. For example, targeted or hybrid consultation approaches [100] could be considered, along with the establishment of clear parameters and processes for responding to feedback [70, 77].  In addition, this study provided a nuanced view of the potential role of evidence as part of policy processes. Although policy makers, advocates and previous policy studies agree on the importance of evidence in obesity prevention policy, the focus of such discussion has largely been on evidence of the policy problem and effectiveness of policy solutions [18, 97, 101]. This study demonstrated the need for policy actors to employ evidence regarding multiple aspects of policy implementation in support of policy proposals. For example, evidence of feasible implementation of policy from other jurisdictions and related contexts was found to be valuable in increasing decision-maker acceptability and support. |
